# Mechanistic investigations reveal that dibromobimane extrudes sulfur from biological sulfhydryl sources other than hydrogen sulfide†
†Electronic supplementary information (ESI) available: Experimental details, pH stability data for BTE, NMR spectra. See DOI: 10.1039/c4sc01875c


**DOI:** 10.1039/c4sc01875c

**Published:** 2014-10-10

**Authors:** Leticia A. Montoya, Xinggui Shen, James J. McDermott, Christopher G. Kevil, Michael D. Pluth

**Affiliations:** a Department of Chemistry and Biochemistry , Institute of Molecular Biology , Materials Science Institute , University of Oregon , Eugene , OR 97403 , USA . Email: pluth@uoregon.edu; b Department of Pathology , Louisiana State University Health Science Center , Shreveport , LA 71130 , USA . Email: ckevil@lsuhsc.edu

## Abstract

Hydrogen sulfide (H_2_S) has emerged as an important biological signaling molecule in the last decade. During the growth of this field, significant controversy has arisen centered on the physiological concentrations of H_2_S. Recently, a monobromobimane (mBB) method has been developed for the quantification of different biologically-relevant sulfide pools. Based on the prevalence of the mBB method for sulfide quantification, we expand on this method to report the use of dibromobimane (dBB) for sulfide quantification. Reaction of H_2_S with dBB results in formation of highly-fluorescent bimane thioether (BTE), which is readily quantifiable by HPLC. Additionally, the reaction of sulfide with dBB to form BTE is significantly faster than the reaction of sulfide with mBB to form sulfide dibimane. Using the dBB method, BTE levels as low as 0.6 pM can be detected. Upon use of the dBB method in wild-type and CSE^–/–^ mice, however, dBB reports significantly higher sulfide levels than those measured using mBB. Further investigation revealed that dBB is able to extract sulfur from other sulfhydryl sources including thiols. Based on mechanistic studies, we demonstrate that dBB extracts sulfur from thiols with α- or β-hydrogens, thus leading to higher BTE formation than from sulfide alone. Taken together, the dBB method is a highly sensitive method for H_2_S but is not compatible for use in studies in which other thiols are present.

## Abstract 2

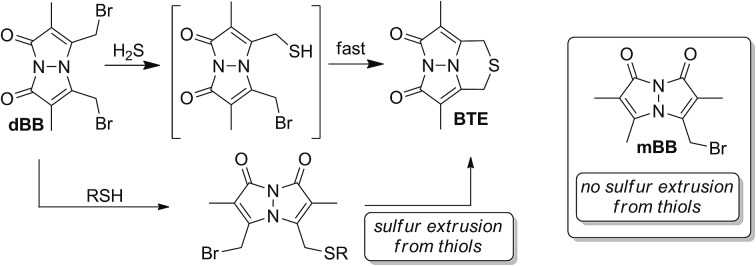
Dibromobimane detects sulfide levels as low as 0.6 pM, but reacts in unexpected ways with thiols, as evidenced by mechanistic investigations.

## Introduction

Hydrogen sulfide (H_2_S) is now recognized as a ubiquitous gaseous signaling molecule that plays important and diverse roles in the endocrine, neuronal, and cardiovascular systems.^1^–^3^ Misregulation of basal H_2_S levels has been demonstrated to be associated with various (patho) physiological diseases ranging from diabetes to hypertension.^1^ Additionally, abnormal H_2_S levels contribute to various disorders of mental deficiency including Alzheimer's disease and Down syndrome.^4^–^6^ Most biological H_2_S is produced enzymatically from cystathionine-β-synthase (CBS), cystathionine-γ-lyase (CSE), and cysteine amino transferase (CAT) working in concert with 3-mercaptosulfurtransferase (3MST), although recent reports have documented H_2_S production for d-cys through a d-amino acid oxidase (DAO) 3MST pathway.^7^ Once generated, H_2_S exists primarily as HS^–^ under physiological conditions, however the accessibility of different protonation states broadens its ability to diffuse across cell membranes, modulate its nucleophilic or reduction potential, and modulate its reactivity with metal targets.^8^–^11^ In addition to its different protonation states, sulfide can be stored in acid-labile sources, such as iron–sulfur clusters, or in partially-oxidized sulfur pools including hydrodisulfides/persulfides (RS-SH), hydropolysulfides (RS_*x*_-SH), and polysulfides (RS-S_*x*_-SR).^12^,^13^ These diverse protonation and storage states not only complicate unravelling the multifaceted biological roles of H_2_S, but also complicate H_2_S detection or quantification.

Despite the widespread and accepted emergence of new biological functions of H_2_S, meaningful forward progress has been slowed in many cases by the dearth of appropriate methods of H_2_S detection and quantification. Although the last few years have seen an impressive growth of new reaction-based methods for H_2_S detection,^14^–^25^ few of these methods are suitable for quantification of endogenous sulfide levels. Most fluorescence-based probes exhibit low micromolar functional detection limits in biological systems, which makes the accurate measurement of real-time H_2_S genesis an unmet challenge.^26^–^30^ Furthermore, although many of these systems show good selectivity for H_2_S over other reactive sulfhydryl-containing species, potential side- or competing-reactions often produce identical products to those generated upon reaction with H_2_S, thus precluding accurate H_2_S quantification in complex samples. This ambiguity, as well as whether such scaffolds report on free, acid-labile,^31^ or total sulfide remains a challenge in further understanding the multifaceted roles of H_2_S.

Direct H_2_S quantification has been maligned by similar challenges. For example, use of the methylene blue method, which was the measurement standard of the field for many years, requires sample acidification followed by treatment with *N*,*N*-dimethyl-*p*-phenylenediamine and FeCl_3_ to generate the methylene blue dye. This method typically reported mid-micromolar levels of H_2_S in biological samples.^32^–^34^ Because the human nose is sensitive to aqueous solutions of 1 μM H_2_S, such results do not match well with qualitative observational data.^34^ Additionally, the reaction conditions required for methylene blue formation, especially treatment with strong acid, can result in liberation of sulfide from acid-labile sulfur sources, such as iron–sulfur clusters.^35^ Furthermore, it has been shown that the methylene blue method is insufficient to differentiate between wild type and heterozygous CSE knock out mice,^36^ and has a revised detection limit of 2 μM, which is much less sensitive than the initially indicated detection limit (∼10 nM). Taking these limitations into account, many of the measured levels of H_2_S have come under increased scrutiny as new, improved methods for H_2_S measurement are developed.

**Fig. 1:**
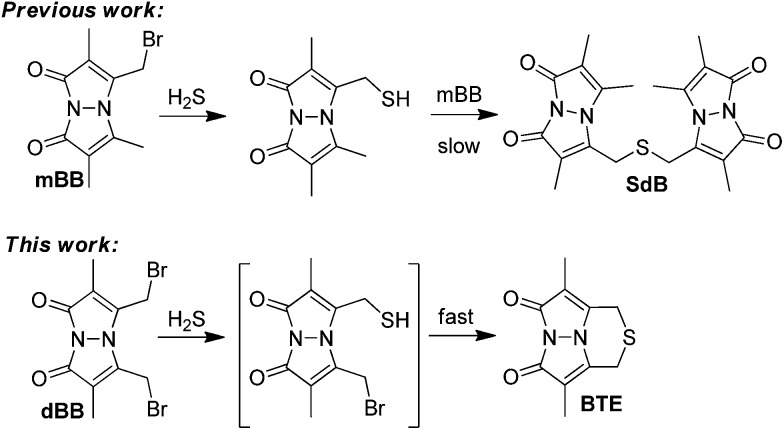
Reaction of mBB and dBB with H_2_S forms the SdB and BTE products, respectively. Both SdB and BTE can be quantified by fluorescence HPLC.

One method that has helped to clarify actual biological H_2_S levels is the monobromobimane (mBB) quantification method.^36^–^38^ In this method, the sample of interest is treated with mBB to trap sulfide as sulfide dibimane (SdB) (Fig. 1). One key benefit of the mBB method is that the analytical selectivity for H_2_S over other thiols can be superimposed at the end of the experiment by chromatographic separation of the different reaction products by HPLC. Additionally, the use of different sample treatment workflows allows for the separation and quantification of free, acid-labile, and total sulfide thereby allowing for direct investigation of different sulfide pools.^37^ With a 2.0 nM detection limit, the mBB method is sensitive enough for most biological applications and has found wide application ranging from clinical to experimental studies investigating sulfide metabolism.^7^,^39^–^43^ Despite this prevalence, several limitations exist, including the high mBB loading required to effectively trap all H_2_S and sulfhydryl nucleophiles, as well as the required trimolecular reaction between H_2_S and two equiv. of mBB. We viewed that use of dibromobimane (dBB), which has two pendant electrophilies on the same fluorogenic platform, would serve as a viable strategy to improve the mBB assay. We report here a full study of mBB and dBB sulfide quantification, which provides unexpected results regarding the sources from which dBB extracts sulfur in biological samples, and provides a detailed mechanistic analysis of the activity of both mBB and dBB in the presence of other thiol reagents.

## Results and discussion

### Comparing the mBB and dBB sulfide response

With the broad use of the mBB method as a sensitive and robust H_2_S quantification method, modifications to this system allowing for faster sulfide trapping and/or lower trapping agent loadings would provide a significant benefit. Because mBB reacts with any sulfhydryl-containing nucleophiles, high concentrations of mBB are required to effectively trap H_2_S in the presence of endogenous thiols. Additionally, because reaction with H_2_S initially generates bimane-SH, sufficient concentrations of mBB must be used such that each bimane-SH produced is efficiently converted to SdB prior to HPLC quantification. To overcome such limitations, we viewed dBB as an attractive platform for enhanced sulfide quantification. Specifically, dBB should react with H_2_S in a 1 : 1 stoichiometry, thus not only improving the reaction kinetics, but also subsequently lowering the overall trapping agent concentration required for effective H_2_S quantification. Recently, we have noted that dibromobimane has been used as a turn-on fluorescent sensor for H_2_S;^44^,^45^ however, this use is problematic because the reaction products of dBB with thiols also generate fluorescent bimane thioether products, thus precluding fluorogenic selectivity for sulfide over thiols without prior chromatographic separation of the fluorescent components.

For both mBB and dBB, the initial attack of HS^–^ to generate bimane-SH should be fast due to the higher acidity of H_2_S by comparison to thiols. For mBB, the generated bimane-SH must undergo a second bimolecular reaction with mBB to form the SdB product. This reaction is inherently slower than the reaction with sulfide due to the decreased nucleophilicity of the bimane sulfhydryl group by comparison to HS^–^. For dBB, however, although the initial attack should proceed at the same rate as for mBB, the subsequent attack of the pendant thiol is now transformed into an intramolecular reaction, thus greatly increasing the potential rate of reactivity. To confirm this design hypothesis, we treated 3.3 mM solutions of mBB and dBB with 3.3 μM H_2_S under the conditions used for the mBB method and compared the rates of reaction by fluorescence spectroscopy (Fig. S1†). As expected, the growth of the fluorescence signal of the BTE product is faster than that of SdB, thus confirming the importance of the intramolecular reaction manifold for maximizing the rate of sulfide trapping.

Having demonstrated that dBB traps H_2_S more quickly than mBB, we next compared the photophysical properties of the SdB and BTE products (Table 1, Fig. S2†). Treatment of either mBB or dBB with NaSH in CH_3_CN/buffer solutions followed by purification afforded the SdB and BTE products in moderate yield. The absorption maxima (*λ*_max_), extinction coefficients (*ε*), emission maxima (*λ*_em_), quantum yield (*Φ*), and brightness (*ε* × *Φ*) were measured for both SdB and BTE and are shown in Table 1. As expected, the extinction coefficient for SdB is larger than that of BTE because two bimane fluorophores are present in the molecule, thus increasing the absorption cross section. Although the emission maxima of SdB and BTE are similar, the quantum yield of BTE (62%) is significantly higher than that of SdB (8.3%). This enhancement is likely due to abolishment of internal quenching mechanisms from the two bimane fluorophores in SdB. Furthermore, comparing the brightness of SdB and BTE, which normalizes the quantum yield to the relative molar absorptivity of each species, reveals that the BTE product is over four times brighter than SdB. These direct comparisons of the photophysical properties of SdB and BTE suggested that detection limit of BTE should be significantly lower than that of SdB due to the greater brightness of the BTE product by comparison to SdB.

**Table 1:** Comparison of the photophysical properties of SdB and BTE^*a*^

	Absorption		Emission		Brightness
	*λ*_max_ (nm)	*ε* (M^–1^ cm^–1^)	*λ*_em_ (nm)	*Φ* ^*b*^ (%)	*Φ* × *ε*
SdB	387	8800 ± 100	478	8.3 ± 0.3	730
BTE	356	4800 ± 100	484	62 ± 2	3000

^*a*^Spectroscopic measurements were performed at least in triplicate in 100 mM KCl and 50 mM PIPES buffer at pH 7.4 at 25.0 °C.

^*b*^Quantum yields are referenced to 1 μM fluorescein (*Φ* = 0.95 in 0.1 M NaOH).

**Fig. 2:**
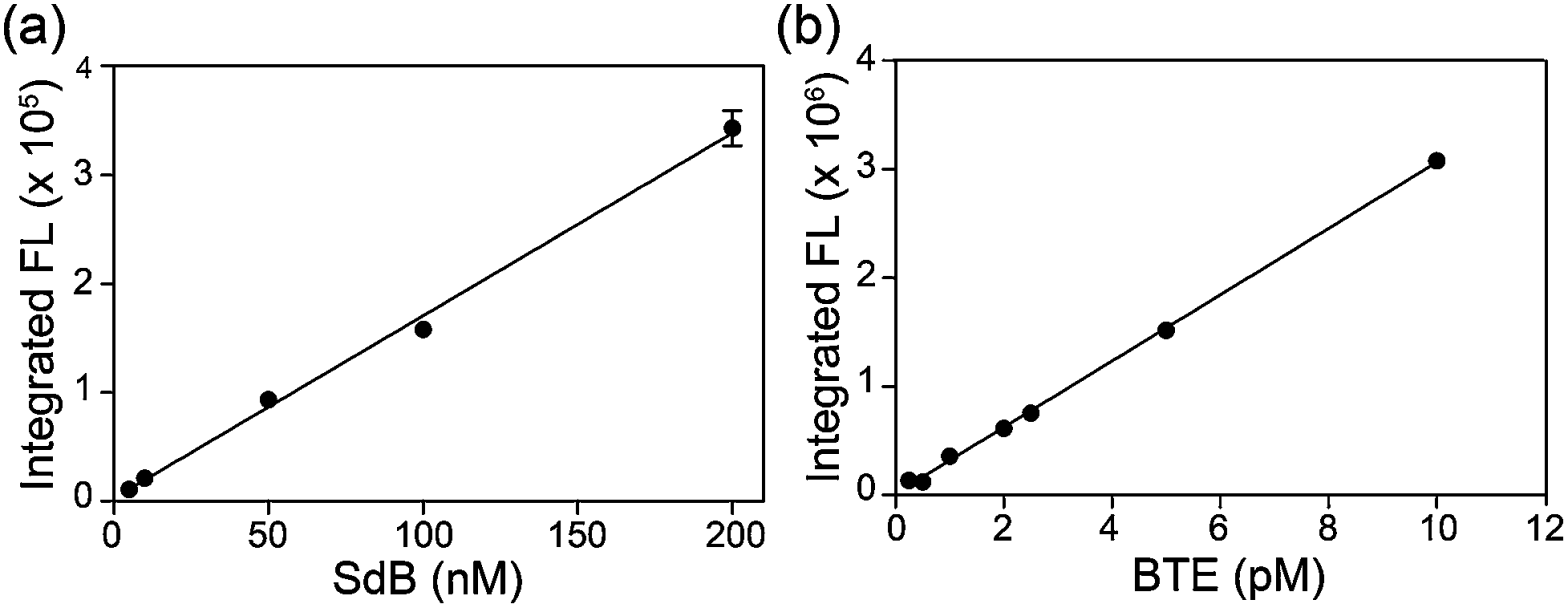
Comparison of the H_2_S detection limits of the mBB and dBB reaction products SdB and BTE, respectively, using fluorescence HPLC.

Based on the photophysical differences between SdB and BTE, we next compared the H_2_S detection limits of mBB and dBB directly. For this comparison, the mBB and dBB reaction products (SdB and BTE, respectively) were compared side-by-side under identical conditions, and on the same instrument used in the initial report of the mBB detection limit. Under these identical conditions, BTE has a superior detection limit by comparison to SdB (Fig. 2). Although SdB provides a 2.0 nM detection limit, which is low enough for most practical biological application of sulfide detection, BTE provides a 0.6 pM detection limit under identical conditions. This detection limit provides a significantly larger window for H_2_S detection and quantification and also opens new avenues of H_2_S detection in which low H_2_S levels are present. To the best of our knowledge, the dBB method provides the most sensitive reaction-based method of H_2_S quantification reported to date.

### Quantification of exogenous and endogenous H_2_S

**Fig. 3:**
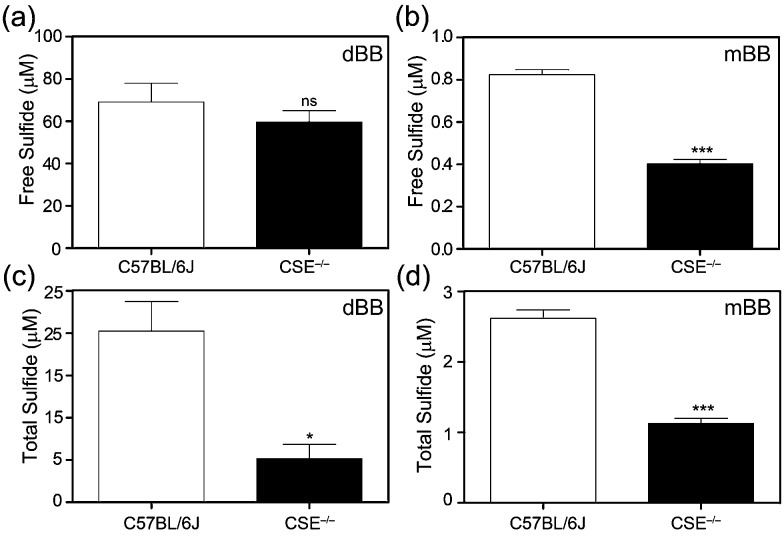
Comparison of free (a and b) and total (c and d) plasma sulfide levels measured using dBB (a and c) and mBB (b and d) in C57BL/6J (WT) and CSE^–/–^ knock out mice. For both mBB and dBB, significant differences are observed in C57BL/6J and CSE^–/–^ mice, however, the absolution sulfide levels quantified are different. ns = non-significant; *p < 0.05, compared to control; ***p < 0.001. *n* = 6 for C57BL/6J, 9 for CSE KO.

To further evaluate the mBB and dBB methods directly, we compared measurements of basal sulfide levels in C57BL/6J (wild type) and CSE^–/–^ (CSE KO) mice. Based on previous work, mBB is sufficiently sensitive to differentiate and quantify differential sulfide levels in the wild type and homozygous CSE knock out mice. Similarly, the mBB method allows for separation of the free, acid labile, and total sulfide pools by either pre-treatment with acid or with a reductant.^37^ For this comparison, both free and total plasma sulfide (free + acid labile + bound sulfur) was quantified using the optimized procedures for the mBB assay from identical samples from the same mice. Based on the results, both mBB and dBB clearly differentiate between the C57BL/6J and CSE^–/–^ mice (Fig. 3). In both cases, however, the quantified sulfide levels were significantly different. The mBB method produced sulfide levels consistent with previous measurements, however the dBB method provided measured sulfide levels that were significantly higher, suggesting that dBB may extract sulfur from other biological sources to which mBB is unreactive. Additionally, the levels of free sulfide measured by dBB are higher than the total sulfide levels, which suggests that other volatile sulfur-containing species that react with dBB, but not mBB, are volatilized in the procedure for free sulfide measurement, this providing another difference between the mBB and dBB methods. Alternatively, the increased BTE formation could also be due to reaction of dBB with proteins in the plasma, such as albumin, which constitutes the majority of thiols in the plasma, or by extrusion of sulfur from circulating sulfane–sulfur species, such as persulfides or polysulfides.^46^

### Comparison of sulfur extrusion by mBB and dBB

**Fig. 4:**
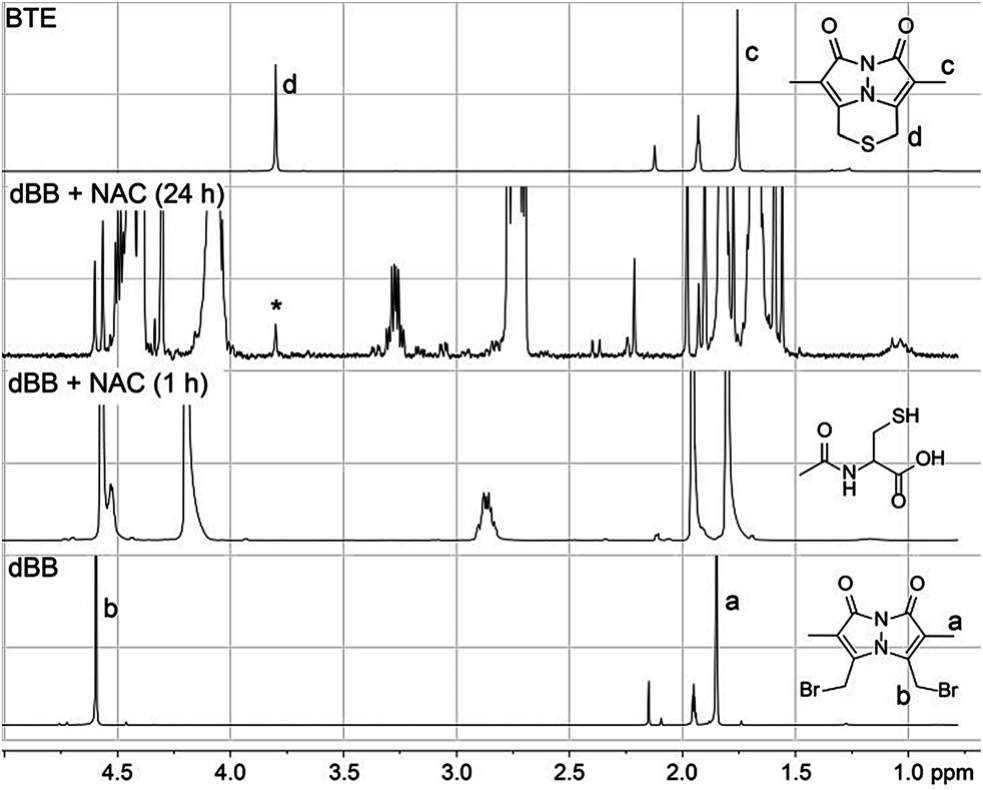
^1^H NMR spectra of the reaction of dBB (50 mM) with *N*-acetyl cysteine (NAC, 20 mM) in CD_3_CN. Growth of a new peak (*) at 3.8 ppm corresponds to the BTE product.

Because both mBB and dBB reported identical sulfide levels when treated with exogenous sulfide sources, we interpreted this result to suggest that dBB was sufficiently reactive to extract sulfur from other sulfur sources, such as thiols. To test this hypothesis, we treated dBB with *N*-acetyl cysteine (NAC) and monitored the reaction by ^1^H NMR spectroscopy. Upon incubation, new ^1^H NMR resonances corresponding to the BTE product were observed in the ^1^H NMR spectrum and were confirmed by the addition of an authentic sample of BTE (Fig. 4). These results suggest that dBB is sufficiently reactive to extrude sulfur from biological thiols to form BTE, thus artificially increasing the measured sulfide levels, which is consistent with the increased BTE formation observed from dBB under biological conditions.

**Fig. 5:**
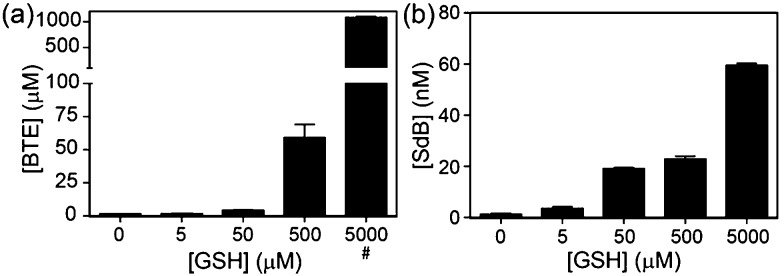
Quantification of sulfur extrusion from GSH by (a) dBB to form BTE and by (b) mBB to form SdB. BTE and SdB concentrations were quantified by HPLC. GSH concentrations were confirmed by reaction with 4-fluoro-7-sulfobenzofurazan (SBD-F) followed by HPLC quantification. Treatment of dBB with 5 mM GSH resulted in detector saturation (#).

To quantify the amount of sulfur extracted from common thiols by dBB, we next investigated and quantified the amount of BTE formed after treatment with reduced glutathione (GSH) and measured the BTE product by HPLC. Consistent with the ^1^H NMR studies, BTE formation was observed by HPLC. To further determine the amount of sulfur extruded from GSH, different concentrations of GSH were added to dBB and the BTE product was quantified by HPLC (Fig. 5). Treatment of mBB with increasing concentrations of GSH ranging from 5 μM to 5 mM only generated low nM concentrations of SdB. By contrast, treatment of dBB with identical GSH concentrations results in generation of micromolar concentrations of BTE. Based on the data, after a 30 minute incubation, dBB extracts approximately 7.0% of the sulfur from GSH to form BTE. By comparison, under identical conditions the mBB method extruded less than 0.01% sulfur from GSH. These extraction efficiencies not only explain the higher levels of biological sulfide detected from dBB but also highlight that mBB does not extract appreciable sulfide from endogenous thiol sources.

### Mechanistic investigations into dBB sulfur extrusion

**Fig. 6:**
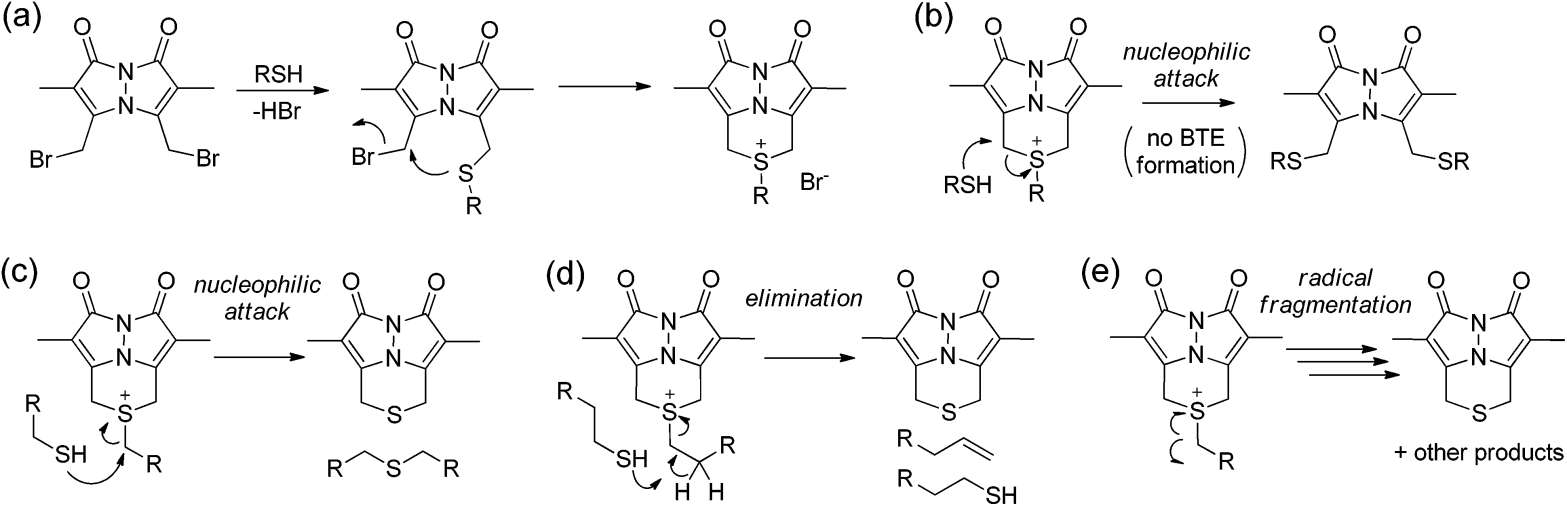
Possible reaction routes of dBB reacting with thiols. (a) Addition of one thiol generates the sulfonium thioether. (b) Nucleophilic addition of a thiol generates the dithiol bimane adduct. Possible mechanisms of BTE formation from thiols, including: (c) nucleophilic attack at the α-position of the pendent thiol; (d) elimination from deprotonation of hydrogens in the β-position of the pendant thiols; and (c) radical fragmentation.

Based on these data, we sought to further investigate the mechanism by which sulfur is extruded by dBB. We viewed three possible mechanisms by which dBB could extract sulfur from thiols (Fig. 6). Each mechanism proceeds through an initial nucleophilic attack of the thiol on one of the electrophilic methylbromide groups to generate the thioether. Subsequent intramolecular attack on the second electrophilic methylbromide would generate the cyclic sulfonium intermediate. From this point, we envisioned three potential mechanisms for dealkylation to form the BTE product. If the sulfonium intermediate maintains a sufficiently unhindered α-position, then nucleophilic attack by a second equivalent of the thiol would generate the BTE product and one equivalent of the thioether derived from the incident thiol (Fig. 6c). Alternatively, if the sulfonium has accessible β-hydrogens, the elimination would extrude the BTE product with concomitant formation of a terminal olefin and regeneration of one equivalent of the incident thiol (Fig. 6d). The third possible mechanism could include radical fragmentation of the sulfonium intermediate to form the BTE product (Fig. 6e).

**Fig. 7:**
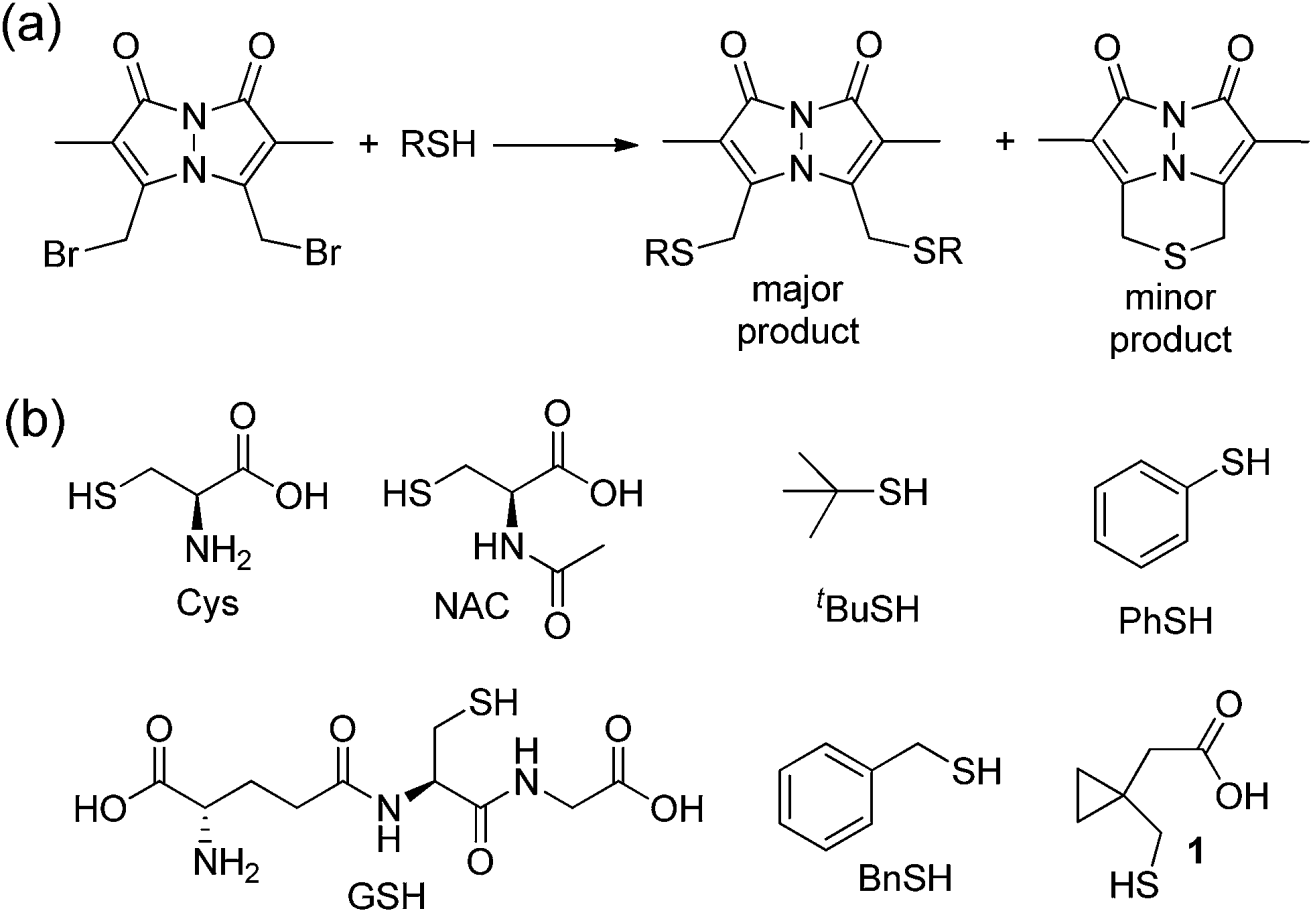
(a) Reaction of dBB with thiols generates either the bis-thioether or the BTE thioether product. (b) Model thiols used to investigate the mechanism by which BTE is formed.

To test between these different mechanistic pathways, we chose multiple model thiols to investigate which pathways of sulfur extrusion were operative and monitored the reactions by ^1^H NMR spectroscopy. In addition to the biologically-relevant cys, NAC, and GSH we also used other thiols to test specific mechanistic considerations (Fig. 7). All of the thiols, except for thiophenol (PhSH), produced the BTE product, which was identified by ^1^H NMR spectroscopy and mass spectrometry.^47^ Because *tert*-butyl thiol generates BTE, we know that nucleophilic attack cannot be the only mechanism of BTE formation because nucleophilic attack on the tertiary carbon is not possible. Similarly, benzyl thiol (BnSH) produced BTE, suggesting that the elimination pathway cannot be the only operative pathway. Consistent with both nucleophilic and elimination pathways leading to BTE formation, treatment of dBB with PhSH, which cannot participate in either of these reaction pathways, failed to produce BTE. If radical fragmentation contributed appreciably to BTE formation, the BTE should have been produced upon treatment with PhSH. To further exclude the radical pathway, we used cyclopropylmethanethiol-containing **1** as a substrate to monitor BTE formation. If the radical pathway were operative, this substrate would generate a methylcyclopropyl radical, which would quickly react (*k* > 10^8^ s^–1^) to the corresponding open-chain product.^48^,^49^ After treatment of dBB with **1** under identical conditions to those of the other thiol substrates, BTE formation was observed but no cyclopropyl ring opening was observed by ^1^H NMR spectroscopy, suggesting that persistent radicals are not formed during the reaction. Similarly, treated dBB with GSH in the presence of DMPO, a radical spin trap,^50^ did not produce any spin-trapped product by EPR spectroscopy. Taken together, these results suggest that both the nucleophilic and elimination pathways are operative in the sulfur extrusion of dBB. Consistent with these results, although BTE is stable at neutral pH, it slowly decomposes in acidic conditions, which is consistent with transient protonation of the thioether sulfur followed by nucleophilic attack by thiol (or solvent) at one of the benzylic bimane carbons (Fig. S3†).

**Fig. 8:**
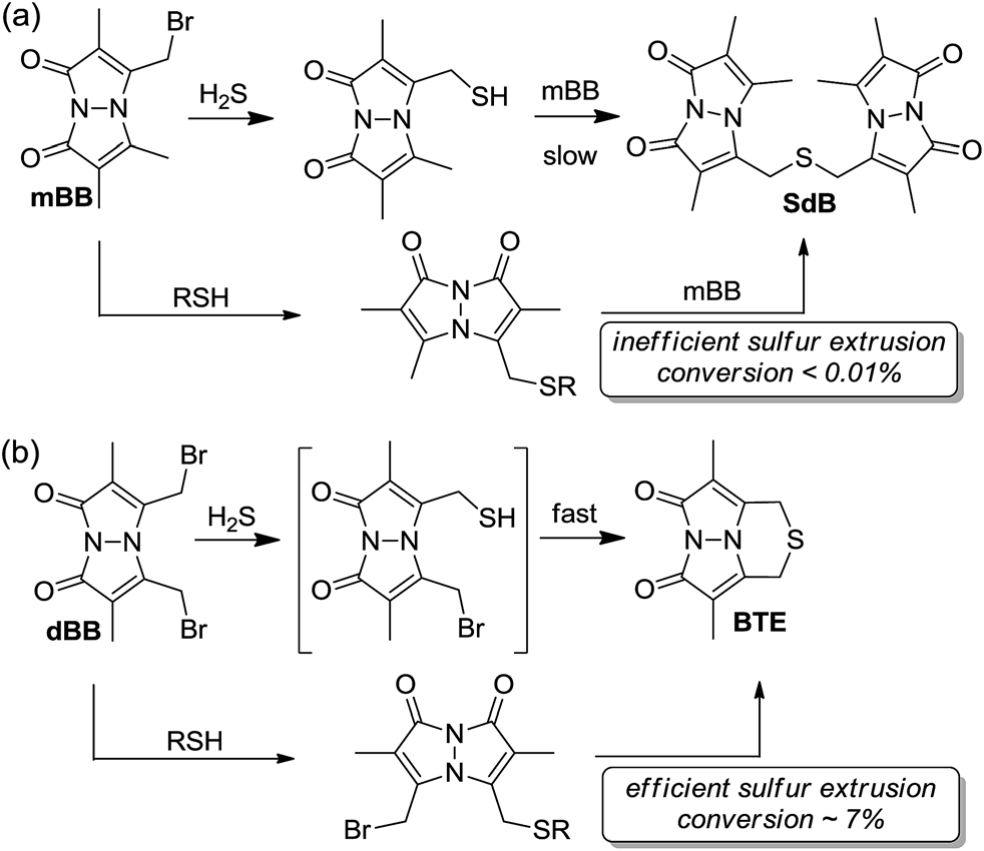
General reaction scheme for (a) mBB and (b) dBB reactivity. Extrusion of sulfur with mBB is inefficient whereas extraction of sulfur with dBB is significantly more efficient.

Comparing the overall reactivity and selectivity reveals that dBB is significantly more sensitive for sulfide than is mBB under conditions without other thiols present. If thiols are present, however, dBB is able to extrude sulfur from these thiols with relatively high efficiency (Fig. 8). In such cases in which thiols can be removed from the sample prior to analysis, dBB provides a highly-sensitive method of H_2_S detection and quantification. For biological samples containing other sulfhydryl containing species, however, mBB is highly efficient for H_2_S quantification. Importantly, mBB very minimally extracts sulfur from thiols, which is not significant, and can be corrected for by measuring total thiol concentrations in a sample.

## Conclusions

Complementing the mBB method, dBB provides a highly-sensitive method for sulfide quantification with a detection limit of 0.6 pM. In the presence of other sulfhydryl containing species, however, dBB extracts sulfur from other sources thereby decreasing its fidelity for H_2_S quantification if other thiols are present. Mechanistic investigations revealed that thiols with α- or β-hydrogens react to generate the BTE product. Taken together, these results establish dBB as a highly-sensitive method for H_2_S quantification, but also provide cautions for its use in biological samples in which thiols are present.

## Supplementary Material

Supplementary information
