# Supplementary material for: Mechanistic investigations reveal that dibromobimane extrudes sulfur from biological sulfhydryl sources other than hydrogen sulfide
Source: Chem Sci. 2014 Oct 10;6(1):294–300. doi: 10.1039/c4sc01875c (PMC4302847; doi:10.1039/c4sc01875c)
Supplement: Supplementary file 1 [file SC-006-C4SC01875C-s001.pdf]

Electronic Supplementary Material (ESI) for Chemical Science.
This journal is © The Royal Society of Chemistry 2014

Supporting Information

Mechanistic Investigations Reveal that Dibromobimane Extrudes Sulfur from Biological Sulfhydryl Sources other than Hydrogen Sulfide

Leticia A. Montoya,[1,a] Xinggui Shen,[2,a] James J. McDermott,[1] Christopher G. Kevil,[2,*] and Michael D. Pluth[1,*]

[1] Department of Chemistry and Biochemistry, Institute of Molecular Biology, Materials Science Institute, University of Oregon, Eugene, OR 97403

[2] Department of Pathology, Louisiana State University Health Science Center, Shreveport, LA 71130

[a] These authors contributed equally to this work

Contact Information:

Michael D. Pluth
pluth@uoregon.edu

Christopher G. Kevil
CKevil@lsuhsc.edu

Table of Contents Page

1. Experimental Details S2
2. Kinetic trace of mBB and dBB reacting with H_2S S6
3. Absorption and emission spectra of SdB and BTE S7
4. Stability of BTE in acid S8
5. BTE Formation from Thiols S9
6. ^{1}H and ^{13}C{^{1}H} NMR spectra of BTE S10
7. References S11

Experimental Details

Materials and Methods

General. NMR spectra were acquired on a Varian INOVA 500 or Bruker 600 MHz spectrometer at 25 °C. Chemical shifts are reported in parts per million (δ) and are referenced to residual protic solvent resonances. Mass spectra were recorded with an Agilent LC/MS mass spectrometer using electrospray ionization in positive ion mode. Fluorescence spectra were obtained on a Photon Technology International (PTI) Quanta Master 40 spectrofluorimeter equipped with a Quantum Northwest TLC-50 temperature controller. UV–visible spectra were acquired on a Cary 100 spectrometer equipped with a Quantum Northwest TLC-42 dual cuvette temperature controller at 25.00 ± 0.05 °C. Molar absorbtivity and quantum yield data were acquired in 50 mM PIPES buffer with 100 mM KCl, pH 7.4, at 25 C in 1.0 cm path length cuvettes obtained from Starna Scientific.

Reagents were purchased from Aldrich or TCI, deuterated solvents were purchased from Cambridge Isotope Laboratories, and mBB and dBB were purchased from Echelon Biosciences. Piperazine-*N*,*N′*-bis(2-ethanesulfonic acid) (PIPES, Aldrich) and potassium chloride (99.999%, Aldrich) were used to make buffered solutions (50 mM PIPES, 100 mM KCl, pH 7.4) with Millipore water. Anhydrous sodium hydrosulfide (NaSH) was purchased from Strem Chemicals and handled under nitrogen. Silica gel (SiliaFlash F60, Silicycle, 230 - 400 mesh) was used for chromatography. Analytical thin-layer chromatography (TLC) was performed on J. T. Baker silica gel plates (250 μm thickness) and preparative TLC was performed on Silicycle SiliaPlates (1 mm thickness). All compounds subjected to preparative chromatography were protected from ambient light during purification.

Plasma collection and processing. C57BL/6J male mice purchased from Jackson labs and CSE knockout mice bred in house were used for this study.[1] Mice were maintained at the association for assessment and accreditation of laboratory animal care international-accredited Louisiana State University Health Science Center-Shreveport animal resource facility and maintained in accordance with the National Research Council's guide for care and use of laboratory animals. All animal studies were approved by the institutional animal care and use committee (protocol P-12-011) and conformed to the guide for the care and use of laboratory animals published by the National Institutes of Health.

Mice were anesthetized by IP injection with 150 mg/kg ketamine and 10 mg/ kg xylazine. Blood was collected into plasma separator tubes from the retro-orbital plexus using heparinized plastic capillary tubes. Plasma samples were immediately placed in a stabilization buffer (degassed 100 mM Tris–HCl buffer, pH 9.5, 0.1 mM diethylenetriaminepentaacetic acid (DTPA)) to preserve bioavailable forms of H_2S and metabolic proteins. Samples were snap-frozen and stored in liquid nitrogen until analyzed.

SdB/BTE HPLC detection method. After derivatization with excess mBB or dBB, concentrations of sulfide dibimane or bimane thioether were measured by Shimadzu Prominence ultra fast liquid chromatograph (HPLC 20A Prominence) equipped with fluorescence detection (λ_{ex} = 390 nm, λ_{em} = 475 nm and λ_{ex} = 358 nm, λ_{em} = 484 nm, respectively) and an eclipse XDB-C18 column (4.6×250 mm, 5 µm). Elution solvents were water containing 0.1% TFA (A) and acetonitrile containing 0.1% TFA (B). For the SdB assay, mobile phase composition (A/B; v/v) was 85:15 at 0 min, 65:35 at 5 min, 45:55 at 16 min, 30:70 at 23 min, 10:90 at 24 min, 10:90 at 26 min and 85:15 at 28 min (isocratic until 30 min). For the BTE assay, mobile phase composition (A/B; v/v) was 90:10 at 0 min, 65:35 at 5 min, 80:20 at 8 min, 40:60 at 16 min, 10:90 at 19 min, 10:90 at 21 min and 90:10 at 22 min (isocratic until 24 min). Both of flow rate were 0.6 mL/min and the injected volumes were 10 µL.

Preparation of calibration standards. The analytical standards were prepared from synthetic and purified SdB.[2] Briefly, 10 µL of 5, 10, 50, 100 and 200 nM SdB or 10 µL of 6.25, 62.5, 125, 250, 500 and 1000 nM BTE were injected into the RP-HPLC. Plotting the peak area of SdB versus the concentration of SdB and performing a linear regression analysis obtained a concentration curve.

General procedure for sulfide measurement. Free and total sulfide of plasma samples were measured with monobromobimane (mBB) as previously described.[1,2] Briefly, blood was collected from mice by retro-orbital bleeding through heparinized plastic capillary tubes directly into BD microtainer plasma separator tubes (NO. 365958). Blood was centrifuged at 3,000 g for 2 min to obtain plasma. For free sulfide measurement, 30 µL of plasma was reacted with 70 µL of 100 mM Tris-HCl buffer (pH 9.5, 0.1 mM DTPA) and 50 µL of 10 mM mBB at 1% oxygen in a hypoxic chamber at room temperature. The reaction was stopped after 30 minutes by adding 50 µl of 200 mM 5-sulfosalicylic acid, and then the sulfide dibimane reaction product was analyzed by a RP-HPLC with an Eclipse XDB-C18 column and fluorescence detection (λ_{ex} = 390 nm, λ_{em}

= 475 nm). For total sulfide measurement, total sulfide is released from 50 μL of plasma by incubating these plasma samples in an acidic solution (pH 2.6, 100 mM phosphate buffer, 0.1 mM DTPA, 1 mM TCEP) in an enclosed system. Volatilized sulfide is then trapped in 100 mM Tric-HCl (pH 9.5, 0.1 mM DTPA) and then reacted with mBB. Sulfide dibimane reaction products were measured by RP-HPLC. Similar to the mBB method, 10 mM of dBB was used to measure free and total sulfide of samples. The reaction product BTE was analyzed by a RP-HPLC with an Eclipse XDB-C18 column and fluorescence detection (λ_{ex} = 358 nm, λ_{em} = 484 nm).

General procedure for SdB and BTE measurements from GSH. A 30 μL aliquot of 0, 5, 50, 500 and 5,000 μM glutathione was added to 70 μL of 100 mM Tris-HCl buffer (pH 9.5, 0.1 mM DTPA) and 50 μL of a 10 mM mBB or dBB stock solution in a hypoxic chamber (1% O_2, room temperature). For mBB samples, 50 μL of 200 mM 5-sulfosalicylic acid was added after 30 minutes incubation. Both mBB and dBB samples were analyzed by RP-HPLC to quantify the formed SdB and BTE, respectively.

General procedure for NMR studies. Stock solutions of dBB (100 mM) and the thiol of interest (40 mM) were prepared in CD_3CN in a glove box. A 500 μL aliquot of the dBB stock solution was transferred to a septum-capped NMR tube and an initial ^{1}H NMR spectrum was obtained. After acquisition of the initial spectrum, 500 μL of the thiol stock solution was added to the NMR tube by syringe and additional NMR spectra were recorded at 1, 2, 4, and 24 hours.

Synthesis

Bimane thioether (BTE). Sodium hydrogen sulfide (8.0 mg, 0.071 mmol) was dissolved in 5 mL of degassed PIPES buffer (pH 9.5) and added drop-wise to a solution of dibromobimane (50.0 mg, 0.071 mmol) in dry, degassed acetonitrile (5 mL). The reaction mixture was stirred under nitrogen overnight in the dark. Unreacted NaSH was removed by purging the reaction mixture with nitrogen for approximately 30 minutes. The resultant reaction mixture was diluted with 5 mL of PIPES buffer and extracted with EtOAc (2 x 40 mL) and CH_2Cl_2 (2 x 40 mL). The combined organic layers were dried with $MgSO_4$ and the solvent was removed under vacuum to afford the crude product, which was purified by SiO_2 chromatography (9:1 CH_2Cl_2:MeOH) to yield the product as a yellow solid (30.7 mg, 97% yield). ^{1}H NMR (300 MHz, CD_3CN) δ: 3.85 (s, 4H), 1.81 (s, 6H, Me). $^{13}C\{^1H\}$ NMR (125 MHz, CD_2Cl_2) δ: 161.27, 150.44, 111.49, 23.19, 6.50. Calcd m/z for [$M+H^+$]: 223.0541, obs. 223.0547.

Sulfide dibimane (SdB). The SdB product was prepared by modification of the published procedure.[2] Sodium hydrogen sulfide (5.6 mg, 0.099 mmol) was dissolved in 5 mL of degassed PIPES buffer (pH 9.5) and added drop-wise to a solution of monobromobimane (50.0 mg, 0.184 mmol) in dry, degassed acetonitrile (2.5 mL). The reaction mixture was stirred under nitrogen for 1 hour in the dark. The crude reaction mixture was diluted with 5 mL of PIPES buffer and extracted with EtOAc (1 x 50 mL) and CH_2Cl_2 (1 x 40 mL). The combined organic layers were dried over $MgSO_4$ and the solvent was removed under vacuum to afford a yellow solid (26.1 mg, 69% yield). ^{1}H NMR (300 MHz, CD_3CN) δ: 3.93 (s, 4H), 2.38 (s, 6H, Me), 1.86 (s, 6H, Me), 1.79 (s, 6H, Me), which matched the previously-reported spectrum.[2]

Kinetic trace of mBB and dBB reacting with H_2S

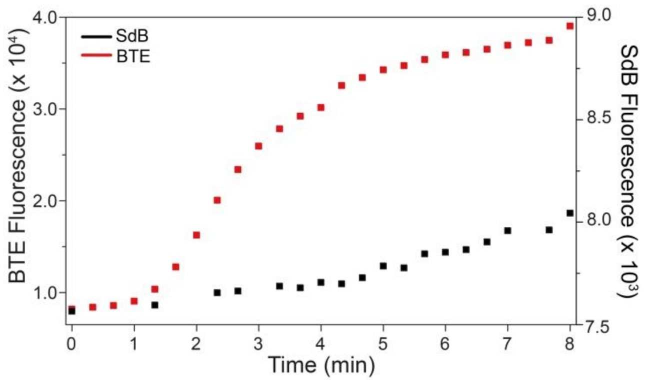

Figure S1. Comparison of the reaction rate of mBB and dBB with H_2S. dBB reacts with H_2S more quickly than mBB. The photophysical properties of BTE make it inherently more emissive than SdB. Conditions: pH 9.5, 50 mM PIPES buffer, 100 mM KCl, 25 °C, 3.3 mM mBB or dBB, 3.3 μM NaSH.

Absorption and emission spectra of SdB and BTE

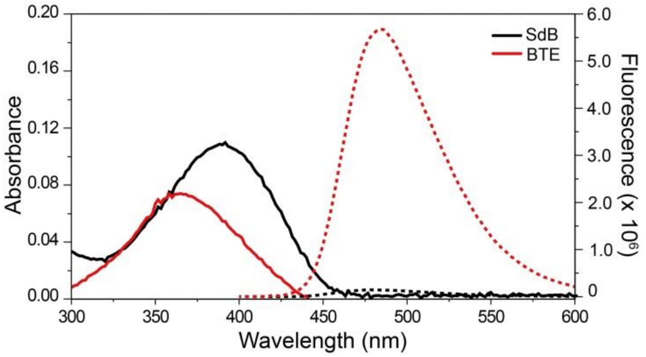

Figure S2. Comparison of the absorption (solid line) and emission profiles (dotted line) of SdB (black) and BTE (red). Conditions: pH 7.4, 50 mM PIPES buffer, 100 mM KCl, 25 °C.

Stability of BTE in acid

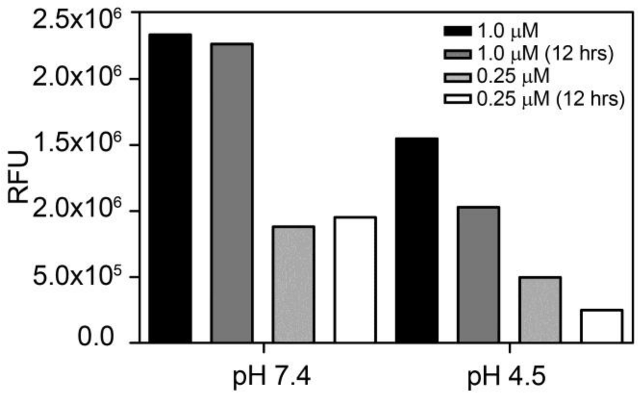

Figure S3. Stability of BTE at pH 7.4 and pH 4.5 after 12 h in buffer. The BTE product was quantified by HPLC.

BTE Formation from Thiols

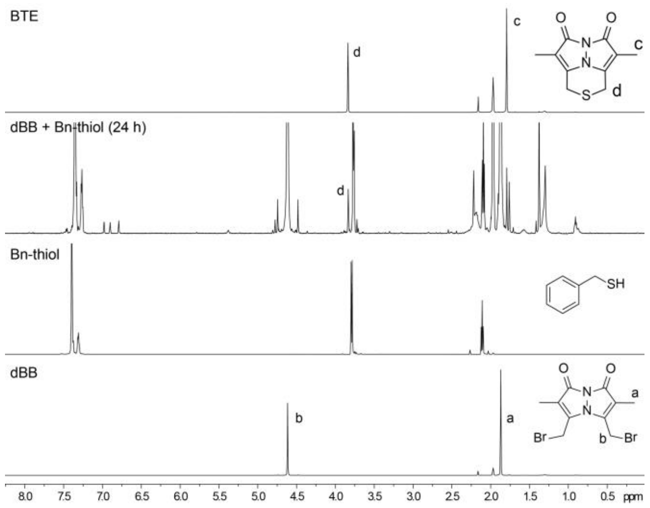

Figure S4. ^{1}H NMR spectra of the reaction of dBB (50 mM) with benzylthiol (20 mM) in CD_3CN. Growth of a new peak (*) at 3.8 ppm corresponds to the BTE product.

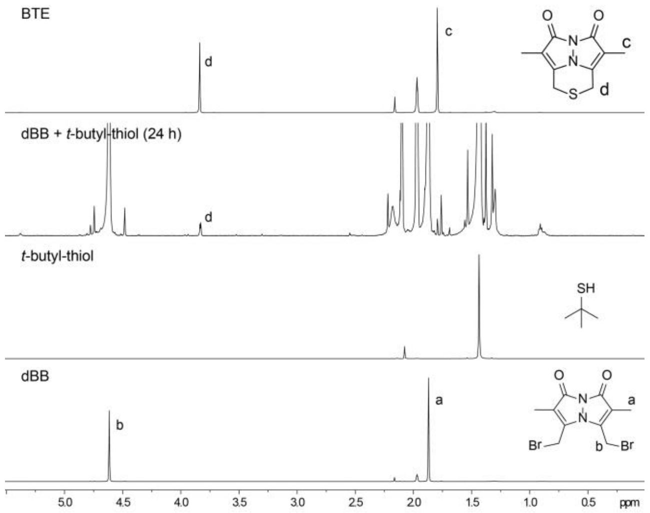

Figure S5. ^{1}H NMR spectra of the reaction of dBB (50 mM) with *tert*-butylthiol (20 mM) in CD_3CN. Growth of a new peak (*) at 3.8 ppm corresponds to the BTE product.

NMR Spectra

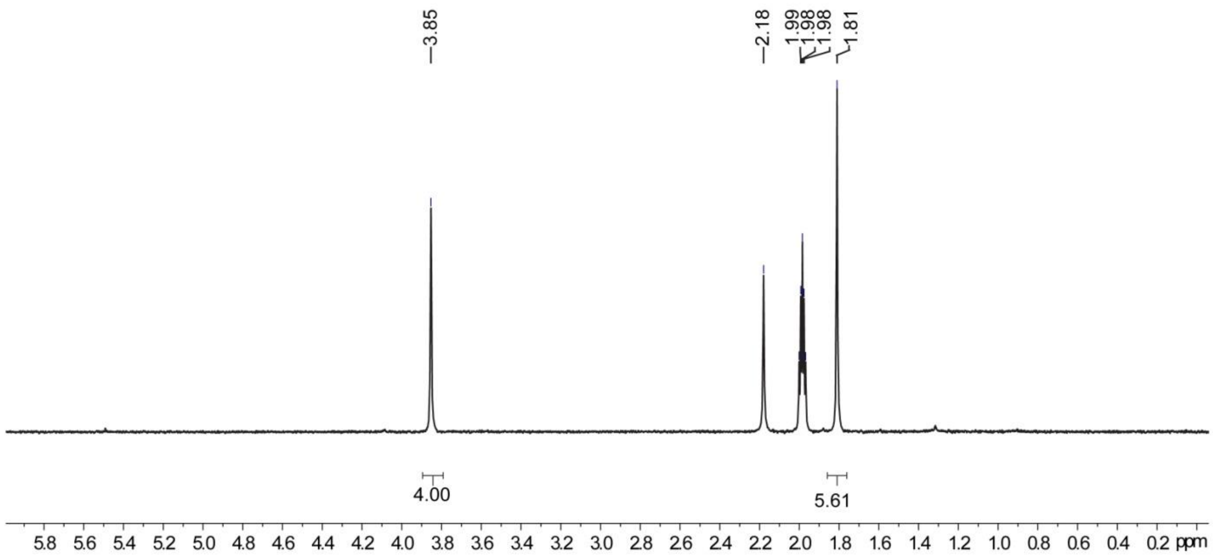

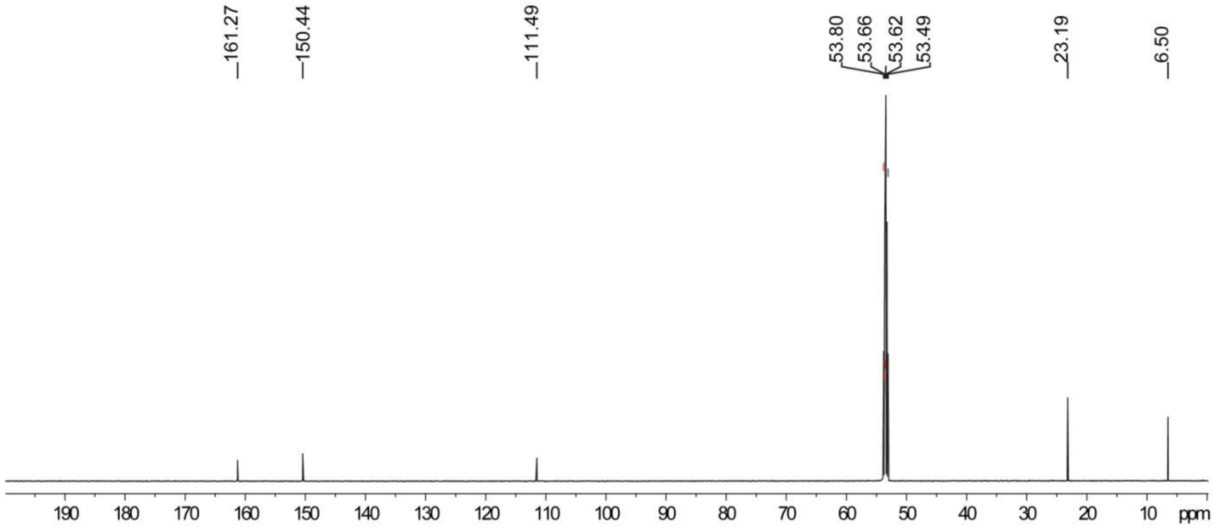

Figure S6. ^{1}H (300 MHz, MeCN) and $^{13}C\{^{1}H\}$ (125 MHz, CD_2Cl_2) NMR spectra of BTE.

References

1. X. G. Shen, E. A. Peter, S. Bir, R. Wang and C. G. Kevil, *Free Radical Biol. Med.*, 2012, **52**, 2276-2283.
2. X. G. Shen, C. B. Pattillo, S. Pardue, S. C. Bir, R. Wang and C. G. Kevil, *Free Radical Biol. Med.*, 2011, **50**, 1021-1031.
